# Systematic literature review and meta-analysis on preventing and controlling norovirus outbreaks on cruise ships, 1990 to 2020: calling for behaviour change strategies of travellers

**DOI:** 10.2807/1560-7917.ES.2024.29.10.2300345

**Published:** 2024-03-07

**Authors:** Varvara A Mouchtouri, Evangelia Simou, Soteris Soteriades, Xanthoula Rousou, Katerina Maria Kontouli, Dimitra Kafetsouli, Leonidas Kourentis, Lemonia Anagnostopoulos, Christos Hadjichristodoulou

**Affiliations:** 1 Laboratory of Hygiene and Epidemiology, Medical School, University of Thessaly, Larissa, Greece; 2 EU SHIPSAN Scientific Association, Larissa, Greece; 3 HEALTHY SAILING project, Larissa, Greece

**Keywords:** Norovirus, gastroenteritis, cruise, infectious disease, maritime, ship, travel, prevention, isolation

## Abstract

**Background:**

Outbreaks of norovirus gastroenteritis aboard cruise ships may affect a large number of people, debilitate vulnerable travellers, disrupt vacations and cause economic losses to the cruise ship industry.

**Aim:**

We aimed to identify risk factors for norovirus outbreaks on cruise ships and assess the effectiveness of prevention and control measures.

**Methods:**

We conducted a systematic literature review searching PubMed and Scopus databases as well as grey literature for articles and reports describing norovirus outbreaks on cruise ships between 1990 and 2020. We also performed a meta-analysis of norovirus prevalence in passengers and crew members.

**Results:**

Data from 45 outbreaks on 26 cruise ships from 1990 to 2020 were identified in 13 articles and five reports, with a weighted average of prevalence (attack rate) for passengers of 7% (95% confidence interval (CI): 5.00–9.00) and for crew of 2% (95% CI: 0.00–3.00). Person-to-person was the most frequent mode of transmission in 35 of the 45 outbreaks (in 14 the only mode and in 21 as part of multiple transmission routes). Having an ill cabin mate (OR = 38.70; 95% CI: 13.51–110.86) was the most common risk factor. Six outbreak investigations reported poor hygiene, while four reported satisfactory hygiene in the cruise setting. Behavioural risk factors among travellers were investigated in three of the 13 studies.

**Conclusions:**

The findings indicate a need for behavioural interventions to improve personal hygiene, symptom reporting and compliance with isolation measures, and for reconsidering current isolation policies where symptomatic and healthy individuals are isolated in the same cabin.

## Introduction

According to the World Health Organization (WHO), gastroenteritis is the most common health problem in travellers [1]. Norovirus has been the leading cause of acute gastroenteritis outbreaks on cruise ships since 2006, although rates of acute gastroenteritis reported among passengers are decreasing [2]. A large study on self-reported illness among travellers found a lower cumulative incidence and declining rates of stomach upset in cruise passengers, compared with other holiday settings [3].

A person can become infected with norovirus indirectly through the ingestion of contaminated food or water, or through contact with contaminated surfaces or objects [4]. Person-to-person transmission occurs either indirectly via the faeces or vomit of infected people (e.g. ingestion of aerosolised vomitus or other aerosolised material resulting from a toilet flush) or directly via contact with an infected individual [4].

Norovirus can be transmitted easily from person-to-person in closed setting such as cruise ships, leading to large outbreaks [5]. To prevent and manage outbreaks on cruise ships, cruise ship companies have developed and implemented outbreak management plans, while governments have established programmes for ship inspections and hygiene standards, including the United States (US) Centers for Disease Control and Prevention (CDC) Vessel Sanitation Programme (VSP) in 1975 and the EU SHIPSAN programme in 2006 [6]. The European Union (EU) project HEALTHY SAILING funded by the Horizon Research and Innovation Action, whose consortium comprises 24 members from 12 countries, has undertaken the task to produce a comprehensive scientific evidence base concerning mechanisms that facilitate the spread of infection and the effectiveness of different mitigation measures on board large passenger vessels [7].

The number of cruise passengers has risen steadily from 13.1 million in 2004 to 28.5 million in 2018, with an average annual growth rate of 5.7% [8-10]. After the pandemic had been declared by the WHO, cruise passenger operations were suspended worldwide. Despite a 66% decline from the already low 2020 levels in 2021, published market reports project cruise bookings to reach pre-pandemic levels by 2024 or 2025 [11].

Epidemiological studies have investigated several norovirus outbreaks in various settings; however, few have examined behavioural aspects of passengers during outbreaks [12,13]. A previous systematic review of norovirus outbreaks on commercial cruise ships highlighted the need for improved outbreak detection and control [14]. However, to our knowledge there is no published study with a systematic appraisal of response measures focusing on behavioural risk factors.

The purpose of this study was to assess prevention and control measures implemented during norovirus outbreaks on cruise ships and to identify risk factors contributing to outbreaks and modes of transmission, by systematically reviewing published outbreak reports. A meta-analysis was conducted to estimate attack rates of norovirus outbreaks and identify risk factors.

## Methods

### Literature review

#### Search strategy

We followed the Preferred Reporting Items for Systematic Reviews and Meta-Analysis (PRISMA) guidelines to conduct the systematic literature review and meta-analysis. We searched PubMed and Scopus databases to identify relevant publications in peer-reviewed journals. The reference lists from relevant articles were searched (hand search), and eligible articles were included in the study. We searched the US CDC VSP website to identify published investigation reports and we sent an official request to receive permission to include investigation reports in the systematic review. We contacted the US CDC VSP since, to our knowledge, it was the only programme that has been conducting specific surveillance and investigation for gastroenteritis outbreaks on cruise ships since 1975, which covered the study period (1990–2020) [15].

We used the following search terms: (gastroenteritis OR diarrhea OR diarrhoea OR illness OR gastrointestinal OR enteritis) AND (ship* OR boat* OR passenger* OR cruise*) AND (outbreak* OR epidemic OR illness OR case* OR cluster ΟR disease) AND (norovirus OR norwalk* OR winter vomiting disease OR Noro*).

Two researchers (VAM and ES) performed the review of the literature independently (for the eligibility criteria and data extraction), and a third reviewer (CH) resolved cases in which there were conflicting views between the two researchers. Initially, the search results were assessed as to whether they met the inclusion criteria by reading the title and abstract. All relevant studies were obtained, and the full text was screened independently by the same reviewers to test eligibility (VAM and ES). For the duplicates that were identified, PubMed was considered the initial reference source.

#### Inclusion and exclusion criteria

The articles that were considered eligible had to report all of the following: (i) laboratory-confirmed norovirus outbreaks on cruise ships, (ii) attack rates among passengers and crew members, (iii) written in English language and (iv) published between 1990 and March 2020. Eligibility criteria regarding the inclusion timeframe were defined to include the period before the COVID-19 pandemic. Another review will be conducted to review gastroenteritis outbreaks after the emergence of COVID-19. Articles reporting norovirus outbreaks on cargo ships or sailing boats, or not meeting any of the other inclusion criteria, were excluded. Outbreaks were considered those that occurred during a cruise and where the cumulative number of cases was greater than what would normally be expected. The threshold for reporting an outbreak was an attack rate of 2% or more for passengers or crew members [15]. Norovirus outbreaks were considered those where faecal samples from at least two infected individuals tested positive for norovirus using reverse transcription PCR (RT-PCR), electron microscopy or enzyme immunoassay [16].

#### Data extraction

We used a pre-designed data extraction form to extract data from studies. Two reviewers (VAM and ES) conducted data extraction independently. The following information was extracted from each outbreak: the time period and cruise itinerary, the causative agent as well as the strain of the agent where available, the number of cases and total sample size of passengers and crew members, the case definition, evidence provided about the mode of transmission, type and details of the epidemiological study conducted, factors examined as contributing to the outbreak, behavioural risk factors, and control measures taken (pre-embarkation screening, surveillance, isolation, cleaning, disinfection, stop sailing, education). We extracted data about the names of norovirus strains as reported by the authors in the primary studies and did not classify strains based on current nomenclature and classification. Outbreaks that occurred on the same ship on subsequent cruises were counted separately as individual subsequent outbreaks. To appraise effectiveness of control measures in the present study, we counted the number of outbreaks in consecutive cruises on the same vessel after the introduction of the control measure. We considered that control measures were not effective on the previous cruise when an outbreak occurred on the subsequent cruise. 

We determined the mode of outbreak transmission and the risk factors after considering the analytical epidemiological results of the outbreak investigation in the original articles. Factors were considered as significantly associated with becoming ill if a p value was < 0.05 in multivariable analysis. The epidemic curve and other risk factors (e.g. ill cabinmate) identified in the primary studies were considered to determine if an outbreak had spread through person-to-person transmission.

### Meta-analysis

We conducted a prevalence meta-analysis with the random-effects model [17], using a log transformation for all studies reporting a single proportion to obtain the estimates. A continuity correction was applied to the studies with zero-cell counts. We used the restricted maximum likelihood (REML). Heterogeneity was explored via the *I^2^* statistic which describes the percentage of variability of effect estimates due to heterogeneity rather than sampling error. We further explored heterogeneity by computing the *Q* statistic as well as the 95% predictive intervals which show the plausible range of effect size values for a future study in another population similar to that included in the metanalysis [18]. We checked for small study effects either visually by inspecting the symmetry of the funnel plot, or statistically by applying Egger’s test if there were more than 10 studies in our analysis. Since heterogeneity was expected to be high, a subgroup analysis based on the cruise itinerary was conducted, to account for some of the observed heterogeneity. In addition, we also present a subgroup analysis based on the different risk factors mentioned as a cause of infection. Results were graphically illustrated in forest plots. All the analyses were performed in R statistical package, version 4.0.3 (R Foundation for Statistical Computing, Vienna, Austria) using the metaprop command of the meta package [19].

All p values of < 0.05 were deemed to represent statistical significance. For the meta-analysis of single proportions, the attack rate was used as the relative measure of effect. Attack rate was defined as the total number of people (passengers or crew members) who became ill, divided by the total number of people at risk of the illness. We estimated attack rate separately for passengers and crew members. Finally, risk factors identified through the literature were included in the meta-analysis.

## Results

### Literature review

**Figure 1:**
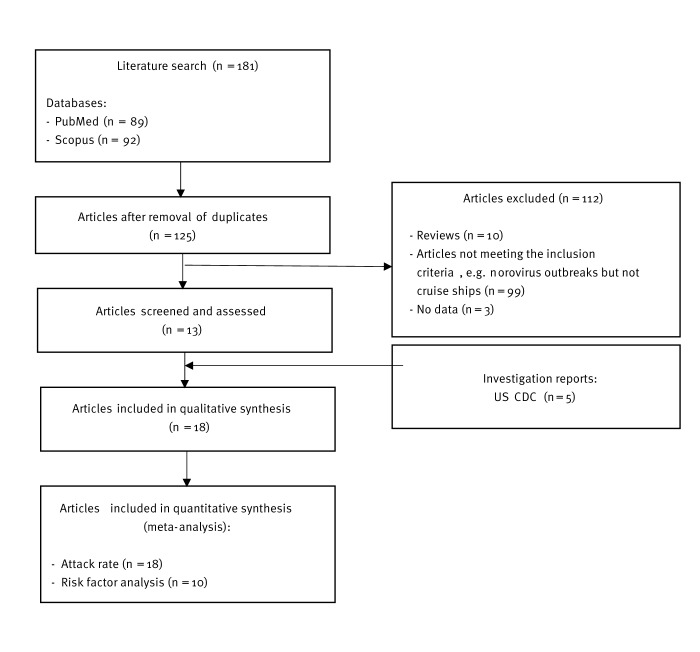
PRISMA flow diagram of the study selection process based on inclusion and exclusion criteria, systematic review on preventing and controlling norovirus outbreaks on cruise ships, 1990–2020 (n = 181)

Of the 181 articles screened, we included 13 articles [13,20-31] in this review (Figure 1). Following the formal request to the US CDC VSP, reports from five investigations conducted in 2006 were shared and included in our review [32]. All studies investigated cruise ship outbreaks with laboratory confirmation of norovirus and the distribution of questionnaires to passengers and crew members. In 11 studies, the authors investigated risk factors by conducting either a case–control study or a cohort study [13,21-24,26-28,30-32]. One study included both types of study design [32]. Moreover, one case–control study was conducted to investigate the hypothesis that consumption of suspected food items in a specific ship kitchen was the source of the outbreak [27]. Behavioural risk factors among travellers were investigated in three of the 13 published studies [13,21,26]. 

US CDC: United States Centers for Disease Control and Prevention.

Data extracted from the 18 studies (13 articles and five reports) concerned 26 cruise ships and a total of 45 outbreaks; however, data on attack rates were available for only 26 cruise ships and 43 outbreaks. Furthermore, only 10 articles included a risk factor analysis related to the mode of transmission. Thirty-three of 43 cruise itineraries took place in the US, Canada, the Caribbean and Mexico, three in Europe, two in China and four in the Pacific Ocean; for one itinerary, data on itinerary were not available. The Caribbean with 20 cruises and Alaska with five of 43 cruises, were the destinations most frequently represented in our sample.

On all 26 cruise ships, norovirus was isolated from faecal samples and in more than half the outbreaks studied, the virus was identified via RT-PCR (23 of 45) [7,13,15,21-24,26-29,31-33]. Besides faecal samples, rectal swabs were taken on one cruise ship, serological tests were taken on two cruise ships in Hawaii, and environmental samples were taken on four cruise ships. Positive environmental samples were found on only one ship [13]. These samples originated from a toilet, the handle of a disinfection container and a restaurant. Overall, the identified strains of norovirus as reported by the authors were: GII.4 Farmington Hills, GI and GII, GII.4, GII, norovirus serotype UK2, GI.3, GII–4 Bristol, GII–4 v6 GU8, GII.4–2006b, GII.4–Minerva, GII.8 and GII.4 (Table 1).

**Table 1:** Attack rate by norovirus strains, cruise ships outbreaks, 1990–2020 (n = 44)

Norovirus strain^a^	**Outbreaks (n)**	**Year of outbreak occurrence**	**Passenger AR**			**Crew member AR**			**Total AR (%)**
			n_infected_	n_total_	%	n_infected_	n_total_	%	
Norovirus^b^	11	2006, 2004, 2002, 1992	1,700	23,448	7.25	102	4,980	2.05	6.34
GII.4 Farmington Hills	11	2002	2,080	22,282	9.33	161	9,536	1.69	7.04
GI and GII	4	2014, 2002	465	6,664	6.98	77	2,433	3.16	5.96
GII.4	3	2002	347	5,859	5.92	38	2,592	1.47	4.56
GII	7	2017, 2002, 1995	206	5,764	3.57	12	1,642	0.73	2.94
Norovirus serotype UK2	2	1990	238	1,151	20.68	NA			20.68
GI.3	1	2002	4	77	5.19	7	35	20.00	9.82
GII–4 Bristol	1	2006	153	1,020	15.00	50	690	7.25	11.87
GII–4 v6 GU8 11690	1	2008	191	1,194	16.00	5	520	0.96	11.44
GII.4–2006b	1	2006	46	98	46.94	2	28	7.14	38.10
GII.4–Minerva	1	2009	236	1,532	15.40	7	859	0.81	10.16
GII.8 and GII.4	1	2002	356	1,984	17.94	13	941	1.38	12.62
**Total**	**44**	**NA**	**6,022**	**71,073**	**8.47**	**474**	**24,256**	**1.95**	**6.48**

AR: attack rate; NA: not applicable.

**^a^** The classification of norovirus strains evolved during the study period and the nomenclature changed, while different variant names were given in the United States and Europe. We extracted names of norovirus strains as reported in the primary studies and did not classify strains based on current nomenclature and classification.

**^b^** No specific strain reported.

Attack rates for each norovirus strain are presented in Table 1. The maximum attack rate for the total ship population, as well as for passengers, occurred in outbreaks caused by the GII.4–2006b [13], followed by norovirus serotype UK2 [22]. However, the most commonly occurring strain was the GII.4 Farmington Hills strain, with 11 outbreaks and an attack rate of 7.04%.

Table 2 presents attack rates by cruise voyage length, which increased as the length of the cruise voyage increased.

**Table 2:** Attack rates by cruise voyage length, norovirus outbreaks on cruise ships, 1990–2020 (n = 38)

Cruise voyage length (days)	**Outbreaks (n)**	**Cruise ships (n)**	**Passenger AR**			**Crew member AR**			**Total AR (%)**
			n_infected_	n_total_	%	n_infected_	n_total_	%	
< 7	3	2	206	5,764	3.57	12	1,642	0.73	2.94
7–10	28	16	3,700	50,284	7.36	357	16,818	2.12	6.05
≥ 11	7	6	1,212	11,440	10.59	99	5,244	1.89	7.86
**Total**	**38**	**24**	**5,118**	**67,488**	**7.58**	**468**	**23,704**	**1.97**	**6.13**

AR: attack rate.

A total of 41 of the 45 outbreaks provided data concerning the source and mode of transmission (Table 3). The most common mode of transmission was person-to-person (35 outbreaks) [21,25,26,28,31,32], either as a single transmission mode (14 outbreaks) or as part of mixed-mode transmission (21 outbreaks). The maximum attack rate (31.71%) occurred in single-mode waterborne transmission [29,30], whereas the minimum attack rate (3.61%) occurred when the mode of transmission was person-to-person and environmental transmission [23,30,31,33]. From the total 31 outbreaks that occurred on subsequent cruises, 27 involved person-to-person transmission, two were food-borne outbreaks and two had an unknown mode of transmission [12,13,20,22,23,25,26,30,32].

**Table 3:** Modes of transmission and attack rates among passengers and crew members, norovirus outbreaks on cruise ships, 1990–2020 (n = 45 outbreaks)

Mode of transmission	**Outbreaks (n)**	**Cruise ships (n)**	**Passenger AR**			**Crew member AR**			**Total AR (%)**
			n_infected_	n_total_	%	n_infected_	n_total_	%	
Person-to-person	14	10	1,715	19,423	8.83	137	15,829	0.87	5.25
Multiple modes^a^	10	5	1,836	12,574	14.60	138	5,364	2.57	11.00
Food-borne	3	2	339	1,706	19.87	NA			19.87
Person-to-person and environmental	11	5	1,134	25,365	4.47	146	10,071	1.45	3.61
Waterborne	3	2	182	515	35.34	14	103	13.59	31.72
Unknown	4	2	493	11,490	4.29	39	2,889	1.35	3.70

AR: attack rate; NA: not applicable.

**^a^** Multiple modes included: (i) person-to-person and multiple exposures (contaminated food, water, surfaces and having a sick cabinmate), (ii) point-source (food-borne), followed by cases associated with person-to-person transmission, (iii) food-borne, person-to-person and environmental, and (iv) waterborne, person-to-person and environmental contamination.

Risk factors identified through the literature for 35 outbreaks are presented in Table 4. These include common exposure at a certain location onboard the ship, consumption of food, consumption of food and common exposure at a ship place, consumption of water, having an ill cabinmate, ill traveller before embarkation, poor hygiene (such as inappropriate food handling, hygiene and storage, no detectable free chlorine in potable water) and travelling with a group before embarkation.

**Table 4:** Risk factors reported to have been significantly associated with norovirus infection on cruise ships after multivariable analysis, and attack rates among passengers and crew members, 1990–2020 (n = 31 outbreaks)

Risk factors	Number of outbreaks (n)	Attack rates (%)			References
		Total	Passengers	Crew members	
Having an ill cabinmate	8	9.87	11.68	2.56	[13,21,26,28,31,32]
Poor hygiene	6	14.41	18.09	2.65	[13,22,24,25,27,31]
Common exposure at a ship place	5	13.50	15.94	2.15	[21,24,26,28,31]
Ill traveller before embarkation	4	6.88	7.55	3.74	[26,32]
Consumption of food	3	30.30	30.85	7.14	[13,22,27]
Consumption of food and common exposure at a ship place	2	12.87	17.95	1.56	[23,30,33]
Consumption of water	2	31.33	32.44	22.83	[13,24]
Travelling with a group before embarkation	1	11.87	15.00	7.25	[32]

A total of 17 of 26 cruise ships reported the control measures that were implemented during outbreaks. The evaluation of these measures was based on the continuation or not of the outbreaks on the subsequent cruise (Table 5). The templates of data collection forms, the data extracted from included studies and data used for all analyses can be found on the website of the HEALTHY SAILING project [4]. In seven of 26 cruise ships, the outbreak continued on the subsequent trip. After introducing the following measures, authors reported that the outbreak did not continue in subsequent cruises: reinforcement of sanitation practices, delaying embarkation for the subsequent cruise, epidemiological investigation, ship removed from service (1 week), cleaning and disinfection, exclusion of ill food handlers from the work place, informative messages to passengers and crew members about hand hygiene, patient isolation, ship inspection, and ship out of service for cleaning and disinfection. It should be noted that the control measures which were considered effective on all cruise itineraries where they were implemented were: reinforcement of sanitation practices and delaying embarkation of the subsequent cruise.

**Table 5:** The control measures that when introduced, authors reported discontinuation of the outbreak, norovirus outbreaks on cruise ships, 1990–2020 (n = 11 outbreaks)

Control measures	**Number of outbreaks before introducing the control measure**	**Attack rate (%)**	**Cruise** **itinerary**	**Reference**
Delaying embarkation of the subsequent cruise Cleaning and disinfection Informative messages to passengers and crew members about hand hygiene Ship inspection	2	2.66	New Jersey Haiti	[32]
Cleaning and disinfection Ship inspection	1	11.87	Caribbean	[32]
Ship inspection Ship out of service for cleaning and disinfection	2	10.42	Alaska	[33]
Reinforcement of sanitation practices Exclusion of ill food handlers from the workplace Ship inspection	1	12.61	Caribbean	[30] [33]
Ship removed from service (1 week) Cleaning and disinfection Ship inspection	4	9.44	Caribbean	[30]
Reinforcement of sanitation practices Cleaning and disinfection Patient isolation	1	3.42	Spain to Florida	[33]

Table 5 presents the six cruise ships (along with their itineraries) that succeeded in having no outbreak on the subsequent cruise. Ship inspection as well as cleaning and disinfection were the most common control measures. The outcome for each control measure that was introduced to stop outbreaks in subsequent cruises (for all ships) is appended in Supplementary Table S1.

### Meta-analysis

We performed a meta-analysis, separately for passengers and crew members, and we observed substantial heterogeneity for both (I^2^ = 99% and 93%, respectively); hence a random-effects model was preferable instead of a fixed-effects model. We also present the fixed effect, as a sensitivity analysis.

**Figure 2:**
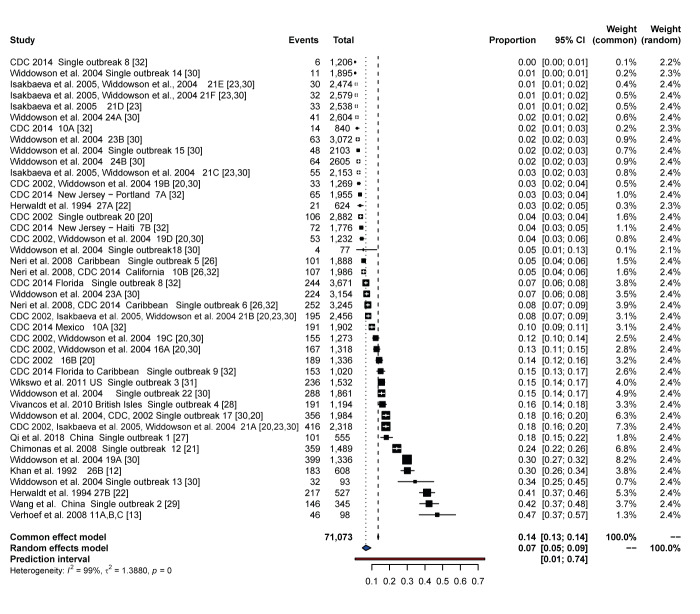
Forest plot of passenger attack rates with 95% confidence intervals, norovirus outbreaks on cruise ships, 1990–2020 (n = 42)

CDC: Centers for Disease Control and Prevention; CI: confidence interval.

**Figure 3:**
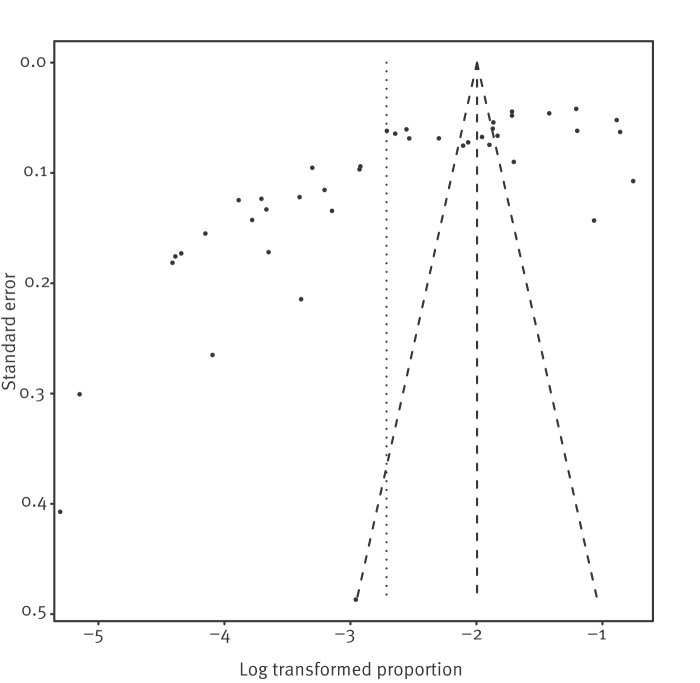
Funnel plot of passenger attack rates, norovirus outbreaks on cruise ships, 1990–2020 (n = 42)

A total of 5,699 persons with confirmed norovirus infection were included out of 71,073 passengers. The weighted proportion of all aetiological agents was 7% (95% CI: 5–9), with substantial heterogeneity among studies (I^2^ = 99%) (Figure 2). Evidence of publication bias (publications in favour of positive results) was found (funnel plot Figure 3 and Egger’s test, p < 0.005).

The number at the end of each reference represents the event (single outbreak on a ship or outbreaks on consecutive cruises on the same ship) and the letter represents each outbreak on the consecutive cruise. Numbers in square brackets refer to the reference list.

The meta-analysis for crew members included a total of 474 persons with confirmed norovirus, out of 24,256 crew members. The weighted proportion of all norovirus infections was 2% (95% CI: 1–3), with substantial heterogeneity among studies (I^2^ = 93%). The proportion of infection for passengers was much higher than for crew members. We found evidence of publication bias (funnel plot (Egger’s test), p = 0.0017). The aforementioned results for crew members are presented also in a forest plot and in a funnel plot in Supplementary Figures S1 and S2, respectively.

Additional subgroup analyses for both passengers and crew members based on cruise itinerary are appended in Supplementary Tables S2 and S3, and Figures S3 and S4. We found subgroup differences for passengers based on the cruise itinerary (p < 0.01); nevertheless, this did not account for the heterogeneity, which remained high for all subgroups, at > 90%.

Subgroup analysis indicated the highest proportion of cases in passengers was found when cruising in China (28%; 95% CI: 12–64) compared with other itineraries (Pacific, Alaska-Canada, Florida-Bahamas-Caribbean and Mexico, and mixed itineraries). However, it should be noted that our systematic literature review included limited data from European cruises. 

An additional subgroup analysis for heterogeneity among crew members has been appended in Supplementary Figure S4 and Table S5, while Supplementary Figures S5 and S6 present additional analyses for risk factors. In a subgroup analysis of crew members based on cruise itineraries, it appeared that there were no differences between groups (p = 0.27). The highest proportion of cases in crew members was 9.00% (95% CI: 0.92–91.5) for cruising in Pacific Ocean; however, this was informed by only two studies and substantial heterogeneity (I^2^ = 93%) was observed.

Passengers who consumed tap water or ice cubes from tap water had a high proportion of infection at 37% (95% CI: 24–58). In addition, consumption of specific food items (one category about ‘food consumption’ as a risk factor was used in the analysis which included all the specific food items that were associated with norovirus infection: cold dishes, cold potherb, cold garlic sprout, cold broad bean, cold cooked egg, carrot pie, whipped cream, fresh-cut fruit and stuffed eggs) appeared to be associated with a higher proportion of cases at 27% (95% CI: 12–61). Crew members who consumed tap water or ice cubes had a higher proportion of infection at 17% (95% CI: 4–66) than all other risk factors we included in the analysis (ill cabinmate, common exposure at a ship place, ill traveller prior to embarkation, poor hygiene, consumption of food).

## Discussion

Our systematic literature review demonstrated that in the vast majority of included norovirus outbreaks, the mode of transmission was person-to-person, either as a single mode or in combination with other transmission modes including food-borne, waterborne and/or environment. Our search strategy identified only three waterborne norovirus outbreaks and after 2002, no waterborne outbreak was identified. Similarly, only a small proportion of the outbreaks reviewed were food-borne outbreaks (five of 41). Our findings confirmed previous studies reporting that the majority of outbreaks on cruise ships are spread from person-to-person, highlighting in particular that cruises lasting more than 7 days provided greater opportunities for person-to-person transmission [2,31]. Food-borne and waterborne outbreaks can be prevented through hygiene measures implemented by crew members on board. Waterborne or food-borne norovirus outbreaks as a result of poor hygiene practices on board have not been published since 2002 (with the exception of two food-borne outbreaks in 2014 and 2017 on river cruises in China); this is probably due to high food and water hygiene standards maintained on cruise ships. Moreover, the construction and operational requirements for water safety were revised in the US CDC VSP manuals in 2001 and 2000 [15]. In the revised versions, additional requirements for monitoring and recording of water disinfection were introduced, as well as requirements for maintenance, monitoring and recording of a cross-connection programme. Another possible explanation could be the publication of inspection results reports on the website, which could have contributed to compliance with standards to avoid bad publicity. 

Most of the included norovirus outbreaks were detected in the US, and this is probably due to the fact that US CDC VSP has been conducting specific syndromic surveillance for gastroenteritis outbreaks since 1975. In other parts of the world, existing specific food-borne and waterborne surveillance schemes can detect such outbreaks on cruise ships, while outbreaks due to person-to-person transmission would not be detected by such schemes. Outbreak detection and reporting processes for cruise ships differ from land-based premises since laboratory capacity for diagnosis is limited on board ships, case definitions used for surveillance are different, and there are limitations in outbreak investigation due to rapid ship movement from country to country [34]. Authorities and researchers might be more likely to publish the results of water- or food-borne outbreaks than of those spread from person-to-person. Moreover, on cruise ships, due to common food sources for the entire travelling population on board, a food-borne outbreak could affect a large number of persons and it is possible that such outbreaks would be more easily detected.

Poor hygiene was reported only in six outbreaks. Previous studies demonstrated that norovirus outbreaks can occur on cruise ships that maintain excellent hygiene standards, including rigorous implementation of prevention and control measures [35]. This is possibly due to the fact that cruise ship hygiene scores cannot reflect the performance of passenger measures for prevention of person-to-person norovirus outbreaks and, as emphasised by Cramer et al., environmental programs cannot “*fully predict and prevent risk factors common to person-to-person and fomite spread of disease*” [36].

Person-to-person transmission can be prevented if passengers and crew apply measures such as handwashing after visiting the toilet, avoiding touching their mouth, handwashing before eating, self-reporting of symptoms and compliance with isolation measures. Only three of our 13 publications examined behavioural issues, an indication that these measures were not adequately studied [13,21,26]. Studies on behavioural risk factors during outbreaks have shown that ill passengers tend to underestimate or ignore gastrointestinal symptoms and do not limit their activities, which may lead to further virus spread [26,31]. Moreover, ill passengers were less likely than healthy passengers to believe that isolation and/or handwashing is effective to prevent disease spread, less likely to wash their hands after restroom use, and less likely to believe that hand sanitiser was available for public use [26,31]. This demonstrates that behavioural risk factors play an important role in the development of an outbreak.

Currently, routine prevention and control measure protocols include syndromic surveillance, pre-embarkation screening, medical diagnosis and care on board, isolation of symptomatic travellers, surface cleaning and disinfection, encouraging personal hygiene measures, crew member education, as well as instructions to travellers and others about handwashing, reporting symptoms and other measures [6,15]. However, recent outbreak reports published on the US CDC website in 2023 (14 outbreaks including 13 related to norovirus and one with *Salmonella* and *Escherichia coli*) underscore the need for different prevention and control approaches [37].

Since prevention and control of person-to-person transmission relies heavily on individual behaviours (performance of personal hygiene, compliance with isolation measures, self-reporting of symptoms), new prevention strategies targeting behavioural change should be introduced, applying principles from behavioural and social sciences. As indicated by the WHO, “*behavioural and social sciences evidence can contribute to and complement other public health efforts that focus on the non-medical factors that influence health outcomes*” [38]. Studying social, cultural or psychological factors about people’s choices and behaviours can help us investigate the reasons behind travellers’ behaviour and better promote and implement preventive policies. We suggest that innovative approaches are needed to develop strategies underpinned by behavioural theory and design appropriate interventions. 

The HEALTHY SAILING project funded by the Horizon Europe programme is expected to produce innovative measures for prevention, mitigation and management of infectious diseases on large passenger vessels, including early detection of threats linked to diseases using artificial intelligence, education for behavioural change in travellers, and improved reporting [7].

The most common risk factor in eight norovirus outbreaks was “having an ill cabinmate” [13,21,26,31,32]. The US VSP currently advises isolation of passengers with acute gastrointestinal symptoms for a minimum of 24 h after symptom resolution; isolation of crew members is required for a minimum of 48 h and 24 h after symptom resolution for employees handling food and other staff, respectively [15]. The *European Manual for Hygiene Standards and Communicable Disease Surveillance on Passenger Ships* recommends that anyone presenting with gastrointestinal symptoms should be isolated (passengers for a minimum of 24 h but preferably 48 hours after symptom resolution, and a minimum of 48 h for crew members handling food and for medical staff) [6]. Isolation measures require separation of ill people from those who are well. However, on fully booked cruises empty cabins may not be available to individually isolate norovirus-infected passengers or crew members, or passengers – especially families – may not be willing to isolate in separate cabins. As a consequence, isolation measures cannot be implemented effectively, leading to continued person-to-person transmission on board, particularly among people staying in the same cabin. Conversely, passengers may not be willing to report symptoms, in order to avoid isolation measures that would disrupt their vacation. Isolation practices and enforcement policies need to be reconsidered, incorporating behavioural science theories and practices.

Early detection and isolation of the first cases is critical to prevent further disease spread [34]. Our review found that in four outbreaks, “ill passenger prior to embarkation” was the common risk factor [26,32]. Previous studies have demonstrated the value of syndromic surveillance for acute gastroenteritis on cruise ships, particularly in cases identified during the first days of the voyage, as well as adverse consequences of reporting delays on the occurrence of norovirus outbreaks on cruise ships [34]. The same study defined that the probability of an outbreak occurring was 11%, if four of 1,000 passengers reported symptoms within the first 2 days of the voyage; this increased to 23% if five of 1,000 passengers reported symptoms within the first 3 days [34].

Our study demonstrated that cruising in the Pacific Ocean (12%; 95% CI: 4–42) and in China (28%; 95% CI: 4–42) was associated with a higher proportion of cases among passengers. This could possibly be explained by exposure of travellers to local community outbreaks and endemic disease (including norovirus) during excursions ashore or consuming products purchased from local markets, without previous assessment of vendors’ food safety standards. Frozen fruits and berries bought from a food vendor caused a norovirus food-borne outbreak on cruise ship sailing in the US [39]. The cruise line had purchased these frozen fruits and berries from a supplier in China. This highlights not only the necessity of purchasing food from approved and previously assessed food suppliers, but the ability to trace the origin of food to quickly recall items not suitable for human consumption.

The meta-analysis showed that the weighted average attack rate for passengers was much higher than for crew members; this confirms previous studies [31,33]. The lower attack rate for crew members could be explained by the limited or lack of direct contact with the passengers [30]. Other factors could be related to immunity or resistance to norovirus infection [31], such as the crew being younger than the passengers, having different exposures or coming from different countries.

Our study has certain limitations. The majority of cruises ship itineraries took place in the Americas (33 of 43) and therefore, the results cannot be considered representative of the global cruise fleet or of cruise destinations worldwide. The meta-analysis was based only on published articles; the estimator of the total effect size might be biased in favour of positive results, since studies with negative results are not reported as often (publication bias). Indeed, in our study, there was evidence of publication bias (Egger’s test). Moreover, while in many studies, the information about the attack rate was determined by a formal recording of all cases, other studies estimated the attack rate by distributing questionnaires. Our review protocol was not registered, but the study protocol is available on the HEALTHY SAILING project website [7]. Heterogeneity tests demonstrated high heterogeneity of the data we included in the systematic literature review. This finding was expected since there were differences in the time and place of data collection in the primary studies in order to estimate prevalence and incidence. In proportion meta-analysis, I^2^ is usually high [40].

## Conclusions

Effective prevention and management of norovirus outbreaks transmitted from person to person is challenging on cruise ships. This is probably due to difficulties in controlling the behavioural factors that influence the implementation of control measures among travellers. Behavioural risk factors among travellers have not been adequately investigated. Study findings urge for behavioural interventions to help improve personal hygiene, early reporting of symptoms and compliance with isolation measures, and for reconsideration of currently implemented isolation policies, where symptomatic and healthy cabinmates are isolated in the same cabin.

## Supplementary Data

363000 Supplement
