# Supplementary material for: Systematic literature review and meta-analysis on preventing and controlling norovirus outbreaks on cruise ships, 1990 to 2020: calling for behaviour change strategies of travellers
Source: Euro Surveill. 2024 Mar 7;29(10):2300345. doi: 10.2807/1560-7917.ES.2024.29.10.2300345 (PMC10986668; doi:10.2807/1560-7917.ES.2024.29.10.2300345)
Supplement: Supplement [file 23-00345_MOUCHTOURI_Supplement.pdf]

This supplementary material is hosted by Eurosurveillance as supporting information alongside the article [A systematic literature review and meta-analysis on preventing and controlling norovirus outbreaks on cruise ships: calling for behaviour change strategies of travellers, 1990-2020], on behalf of the authors, who remain responsible for the accuracy and appropriateness of the content. The same standards for ethics, copyright, attributions and permissions as for the article apply. Supplements are not edited by Eurosurveillance and the journal is not responsible for the maintenance of any links or email addresses provided therein

Table S1 Outbreaks in subsequent cruises after the introduction of control measures

Control measures reported by authors	Outbreaks (n) that the control measure was reported	Multiple control measures reported by authors	Outbreak continued in multiple cruises
**Reinforcement of sanitation practices**	2	Yes	No: 2
**Active case finding**	1	Yes	Unknown: 1
**Delaying embarkation in the subsequent cruise**	1	Yes	No: 1
**Disembarkation of ill people**	3	Yes	Unknown: 2<br>Yes: 1
**Hospitalization of sick ashore**	1	Yes	Unknown: 1
**Ship removed from service (1 week)**	1	Yes	No: 1
**Cessation of self-service buffet**	4	Yes	Unknown: 3<br>Yes: 1
**Cleaning and disinfection**	19	Yes	Unknown: 9<br>Yes: 6<br>No: 4
**Closing down recreational water facilities**	3	Yes	Unknown: 2<br>Yes: 1
**Closing other facilities**	4	Yes	Unknown: 3<br>Yes: 1
**Exclusion of ill food handlers from the work place**	2	Yes	Unknown: 1<br>No: 1
**Food disposal**	4	Yes	Unknown: 2<br>Yes: 2
**Hyperchlorination of potable water**	1	Yes	Yes: 1
**Informative messages to passengers & crew members about hand hygiene**	5	Yes	Unknown: 2<br>Yes: 2<br>No: 1
**Ice discharged**	1	Yes	Yes: 1
**Patient isolation**	8	Yes	Unknown: 5

This supplementary material is hosted by Eurosurveillance as supporting information alongside the article [A systematic literature review and meta-analysis on preventing and controlling norovirus outbreaks on cruise ships: calling for behaviour change strategies of travellers, 1990-2020], on behalf of the authors, who remain responsible for the accuracy and appropriateness of the content. The same standards for ethics, copyright, attributions and permissions as for the article apply. Supplements are not edited by Eurosurveillance and the journal is not responsible for the maintenance of any links or email addresses provided therein

			Yes: 2<br>No: 1
**Ship inspection**	18	Yes	Unknown: 9<br>Yes: 4<br>No: 5
**Ship out of service for cleaning and disinfection**	5	Yes	Unknown: 2<br>Yes: 2<br>No: 1
**Unknown**	16	No	Unknown: 8<br>Yes: 8

This supplementary material is hosted by Eurosurveillance as supporting information alongside the article [A systematic literature review and meta-analysis on preventing and controlling norovirus outbreaks on cruise ships: calling for behaviour change strategies of travellers, 1990-2020], on behalf of the authors, who remain responsible for the accuracy and appropriateness of the content. The same standards for ethics, copyright, attributions and permissions as for the article apply. Supplements are not edited by Eurosurveillance and the journal is not responsible for the maintenance of any links or email addresses provided therein

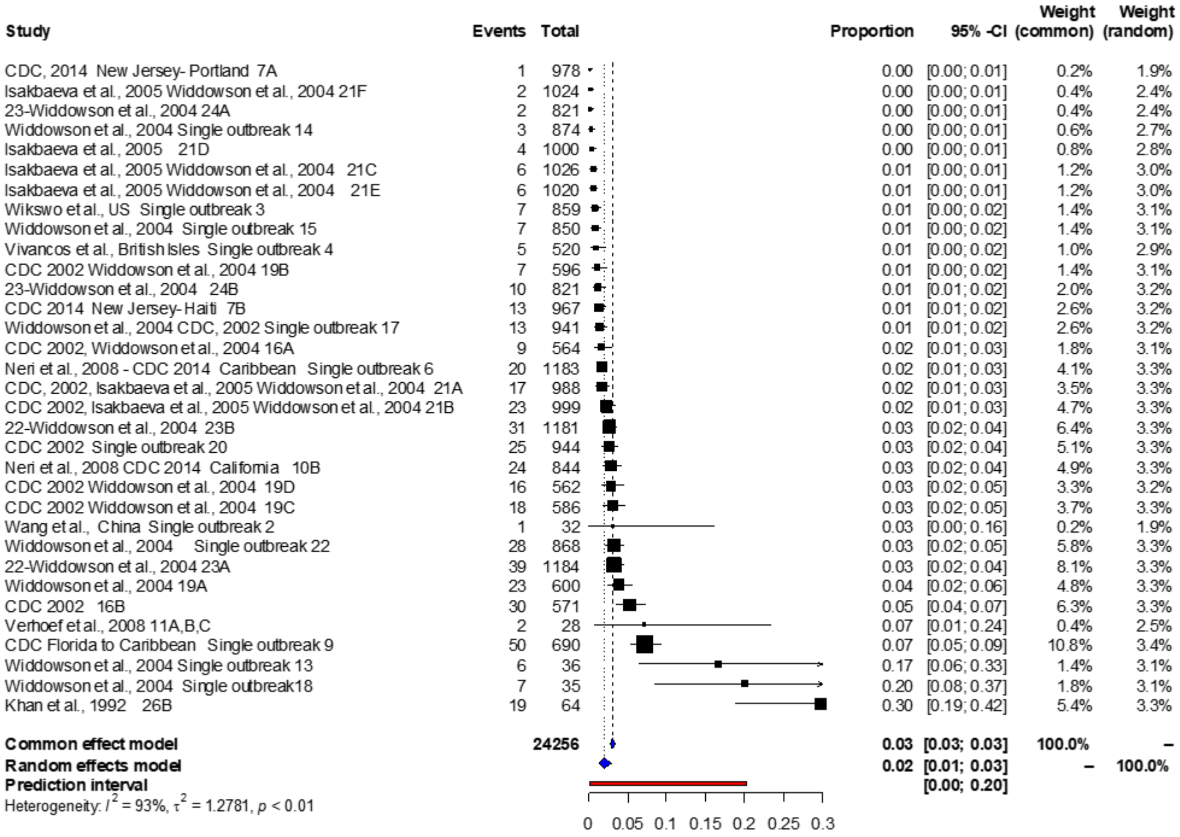

Figure S1: Forest plot: Crew attack rates with 95% confidence intervals

This supplementary material is hosted by Eurosurveillance as supporting information alongside the article [A systematic literature review and meta-analysis on preventing and controlling norovirus outbreaks on cruise ships: calling for behaviour change strategies of travellers, 1990-2020], on behalf of the authors, who remain responsible for the accuracy and appropriateness of the content. The same standards for ethics, copyright, attributions and permissions as for the article apply. Supplements are not edited by Eurosurveillance and the journal is not responsible for the maintenance of any links or email addresses provided therein

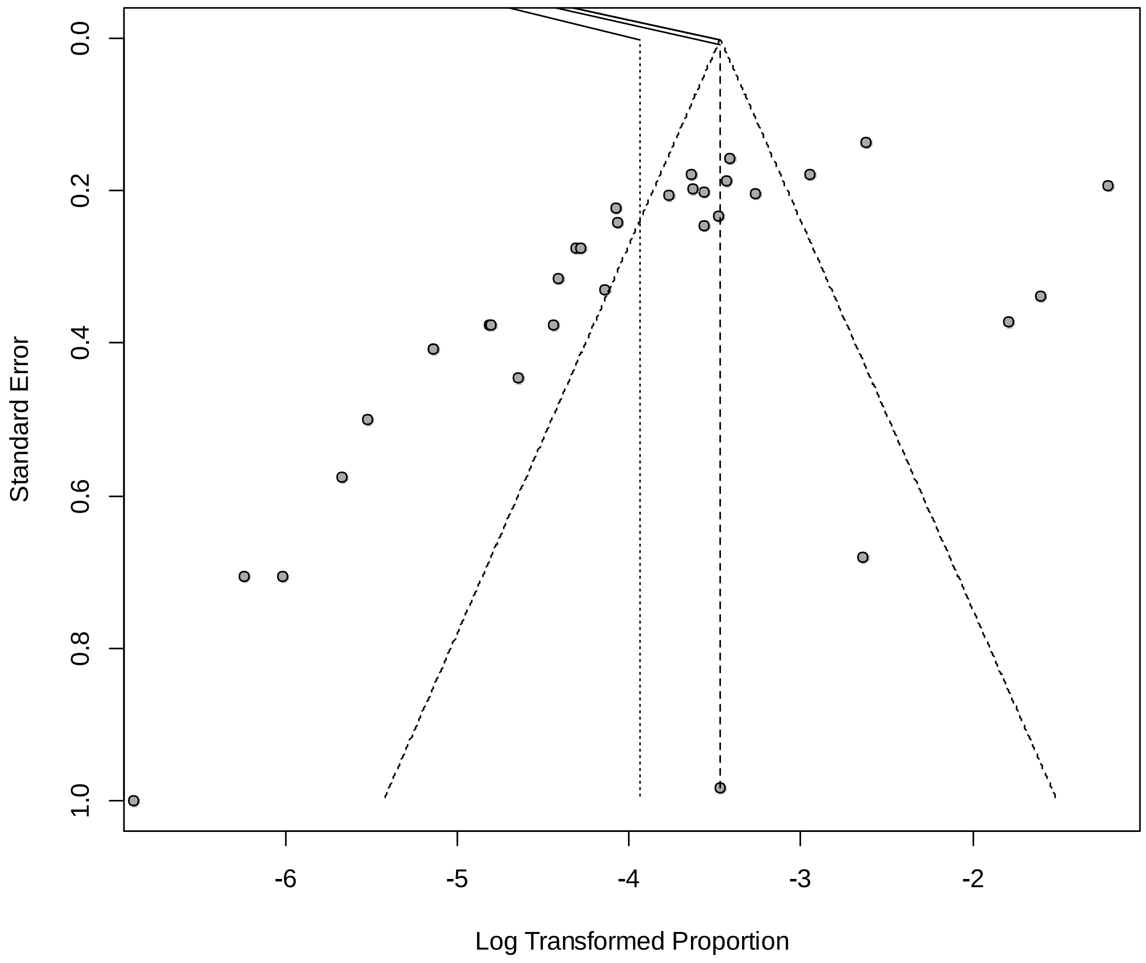

Supplementary Figure S2: Funnel plot: Crew attack rates

This supplementary material is hosted by Eurosurveillance as supporting information alongside the article [A systematic literature review and meta-analysis on preventing and controlling norovirus outbreaks on cruise ships: calling for behaviour change strategies of travellers, 1990-2020], on behalf of the authors, who remain responsible for the accuracy and appropriateness of the content. The same standards for ethics, copyright, attributions and permissions as for the article apply. Supplements are not edited by Eurosurveillance and the journal is not responsible for the maintenance of any links or email addresses provided therein

Table S2 Results of subgroup analysis per cruise itineraries of proportion meta-analysis of passengers

Subgroup	Number of cruises	Proportion	95%-CI	tau^2	I^2
**China**	2	0.278	[0.122, 0.636]	0.350	98.30%
**Mixed itineraries**	5	0.102	[0.043, 0.245]	0.970	98.60%
**Florida-Bahamas-Caribbean-Mexico**	25	0.056	[0.031, 0.070]	1.056	99.00%
**Pacific Ocean**	4	0.123	[0.037, 0.416]	1.517	99.30%
**Europe**	1	0.469	[0.380, 0.579]	NA	N/A
**Alaska-Canada**	5	0.057	[0.015, 0.200]	2.323	99.00%

This supplementary material is hosted by Eurosurveillance as supporting information alongside the article [A systematic literature review and meta-analysis on preventing and controlling norovirus outbreaks on cruise ships: calling for behaviour change strategies of travellers, 1990-2020], on behalf of the authors, who remain responsible for the accuracy and appropriateness of the content. The same standards for ethics, copyright, attributions and permissions as for the article apply. Supplements are not edited by Eurosurveillance and the journal is not responsible for the maintenance of any links or email addresses provided therein

Table S3 Results of subgroup analysis per cruise itineraries of proportion meta-analysis of crew members

Subgroup	k	Proportion	95%-CI	tau^2	I^2
**China**	1	0.031	[0.0045, 0.2151]	NA	NA
**Mixed itineraries**	5	0.013	[0.0028, 0.0568]	2.6515	92.20%
**Florida-Bahamas-Caribbean-Mexico**	20	0.018	[0.0118, 0.0287]	0.9201	89.80%
**Pacific Ocean**	2	0.092	[0.0092, 0.9159]	2.7123	98.60%
**Europe**	1	0.071	[0.0188, 0.2716]	NA	NA
**Alaska-Canada**	4	0.013	[0.0044, 0.0411]	1.1672	92.40%

This supplementary material is hosted by Eurosurveillance as supporting information alongside the article [A systematic literature review and meta-analysis on preventing and controlling norovirus outbreaks on cruise ships: calling for behaviour change strategies of travellers, 1990-2020], on behalf of the authors, who remain responsible for the accuracy and appropriateness of the content. The same standards for ethics, copyright, attributions and permissions as for the article apply. Supplements are not edited by Eurosurveillance and the journal is not responsible for the maintenance of any links or email addresses provided therein

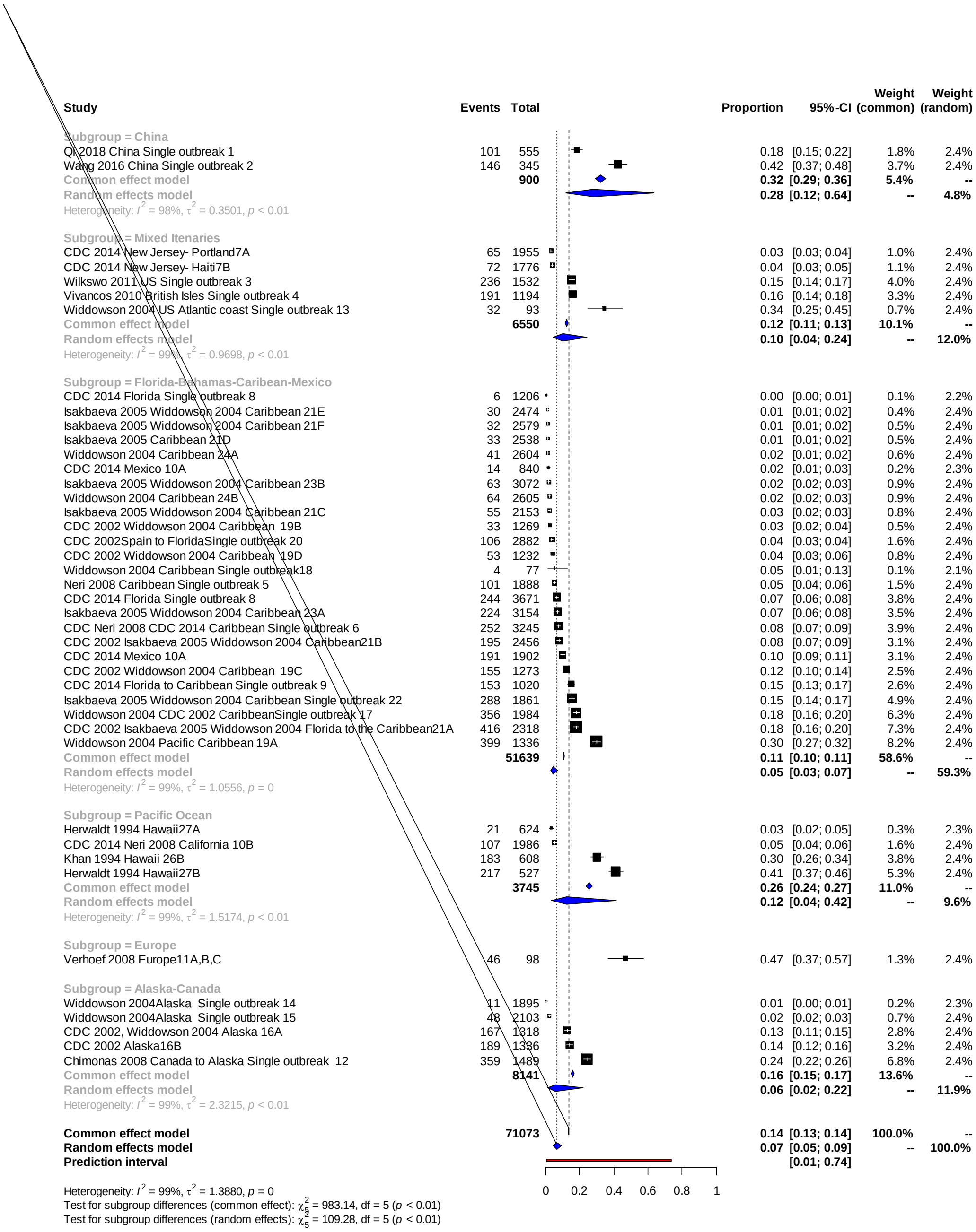

Figure S3: Forest plot: Passenger attack rates with 95% confidence intervals, according to the cruise itinerary

This supplementary material is hosted by Eurosurveillance as supporting information alongside the article [A systematic literature review and meta-analysis on preventing and controlling norovirus outbreaks on cruise ships: calling for behaviour change strategies of travellers, 1990-2020], on behalf of the authors, who remain responsible for the accuracy and appropriateness of the content. The same standards for ethics, copyright, attributions and permissions as for the article apply. Supplements are not edited by Eurosurveillance and the journal is not responsible for the maintenance of any links or email addresses provided therein

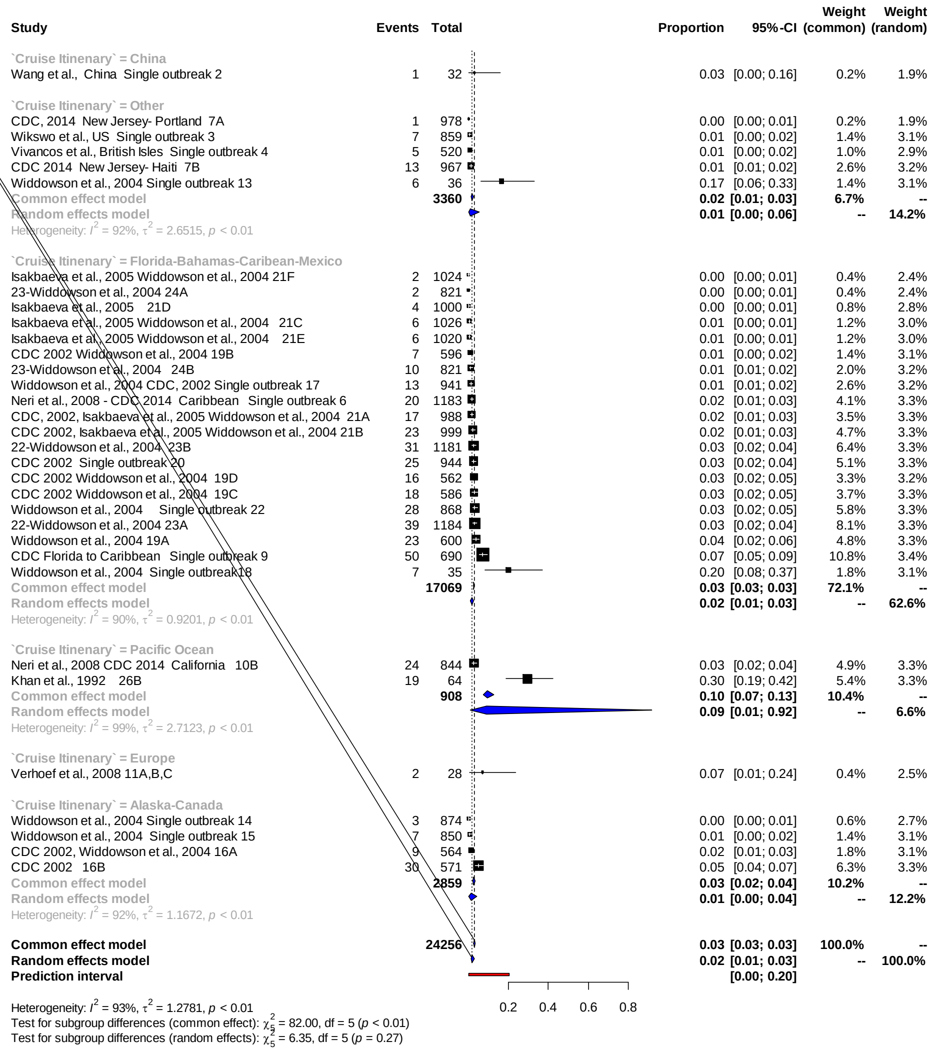

Figure S4: Crew attack rates with 95% confidence intervals, according to the cruise itinerary

This supplementary material is hosted by Eurosurveillance as supporting information alongside the article [A systematic literature review and meta-analysis on preventing and controlling norovirus outbreaks on cruise ships: calling for behaviour change strategies of travellers, 1990-2020], on behalf of the authors, who remain responsible for the accuracy and appropriateness of the content. The same standards for ethics, copyright, attributions and permissions as for the article apply. Supplements are not edited by Eurosurveillance and the journal is not responsible for the maintenance of any links or email addresses provided therein

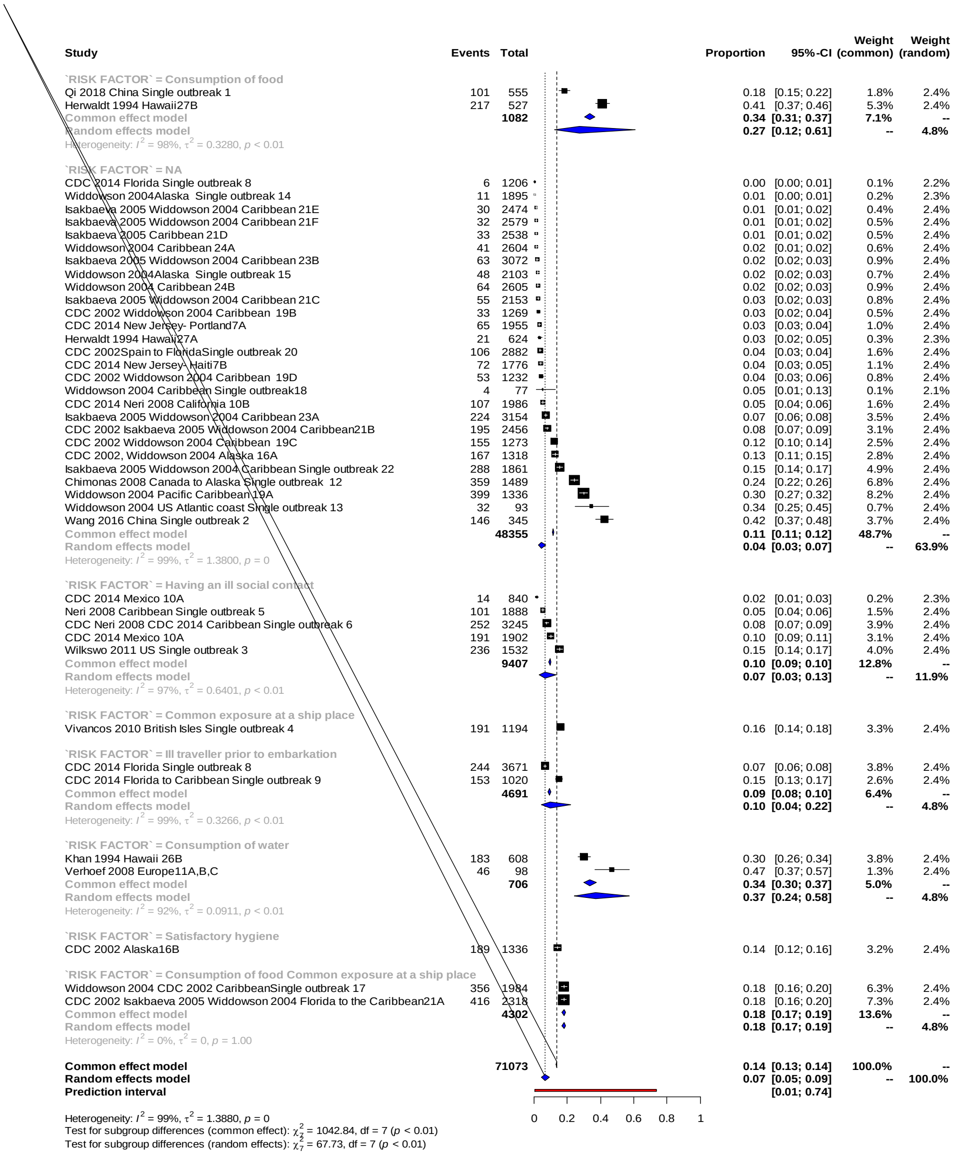

Figure S5: Forest plot: Subgroup analysis for passengers' risk factors, proportions with their corresponding 95% confidence intervals, associated with Norovirus gastroenteritis on cruise ships

This supplementary material is hosted by Eurosurveillance as supporting information alongside the article [A systematic literature review and meta-analysis on preventing and controlling norovirus outbreaks on cruise ships: calling for behaviour change strategies of travellers, 1990-2020], on behalf of the authors, who remain responsible for the accuracy and appropriateness of the content. The same standards for ethics, copyright, attributions and permissions as for the article apply. Supplements are not edited by Eurosurveillance and the journal is not responsible for the maintenance of any links or email addresses provided therein

Table S4 Results of subgroup analysis per risk factors of proportion meta-analysis of passengers

Risk Factor	Number of cruises	Proportion	95%-CI	tau^2	I^2
**Consumption of food**	2	0.2746	[0.1234, 0.6113]	0.328	98.40%
**N/A**	27	0.0444	[0.0284, 0.0695]	1.38	99.20%
**Ill cabinmate**	5	0.0656	[0.0323, 0.1336]	0.6401	97.40%
**Common exposure at a ship place**	1	0.16	[0.1405, 0.1822]	N/A	N/A
**Ill traveler prior to embarkation**	2	0.0997	[0.0449, 0.2215]	0.3266	98.60%
**Consumption of water**	2	0.3726	[0.2412, 0.5758]	0.0911	92.20%
**Poor hygiene**	1	0.1415	[0.1240, 0.1614]	N/A	N/A
**Consumption of food Common exposure at a ship place**	2	0.1795	[0.1683, 0.1913]	0	0.00%

This supplementary material is hosted by Eurosurveillance as supporting information alongside the article [A systematic literature review and meta-analysis on preventing and controlling norovirus outbreaks on cruise ships: calling for behaviour change strategies of travellers, 1990-2020], on behalf of the authors, who remain responsible for the accuracy and appropriateness of the content. The same standards for ethics, copyright, attributions and permissions as for the article apply. Supplements are not edited by Eurosurveillance and the journal is not responsible for the maintenance of any links or email addresses provided therein

Table S5 Results of subgroup analysis per risk factors of proportion meta-analysis of crew members

**Risk factors**	**Number of cruises**	**Proportions**	**95%-CI**	**tau^2**	**I^2**
**N/A**	24	0.0163	[0.0102; 0.0260]	1.2046	87.50%
**Ill cabinmate**	2	0.0125	[0.0062; 0.0253]	0.1709	64.20%
**Common exposure at a ship place**	1	0.0096	[0.0040; 0.0230]	N/A	N/A
**Ill traveler prior to embarkation**	1	0.0725	[0.0555; 0.0946]	N/A	N/A
**Consumption of water**	2	0.1692	[0.0432; 0.6623]	0.7641	75.3%
**Poor hygiene**	1	0.0525	[0.0371; 0.0744]	N/A	N/A
**Consumption of food Common exposure at a ship place**	2	0.0156	[0.0110; 0.0223]	0.000	0.00%

This supplementary material is hosted by Eurosurveillance as supporting information alongside the article [A systematic literature review and meta-analysis on preventing and controlling norovirus outbreaks on cruise ships: calling for behaviour change strategies of travellers, 1990-2020], on behalf of the authors, who remain responsible for the accuracy and appropriateness of the content. The same standards for ethics, copyright, attributions and permissions as for the article apply. Supplements are not edited by Eurosurveillance and the journal is not responsible for the maintenance of any links or email addresses provided therein

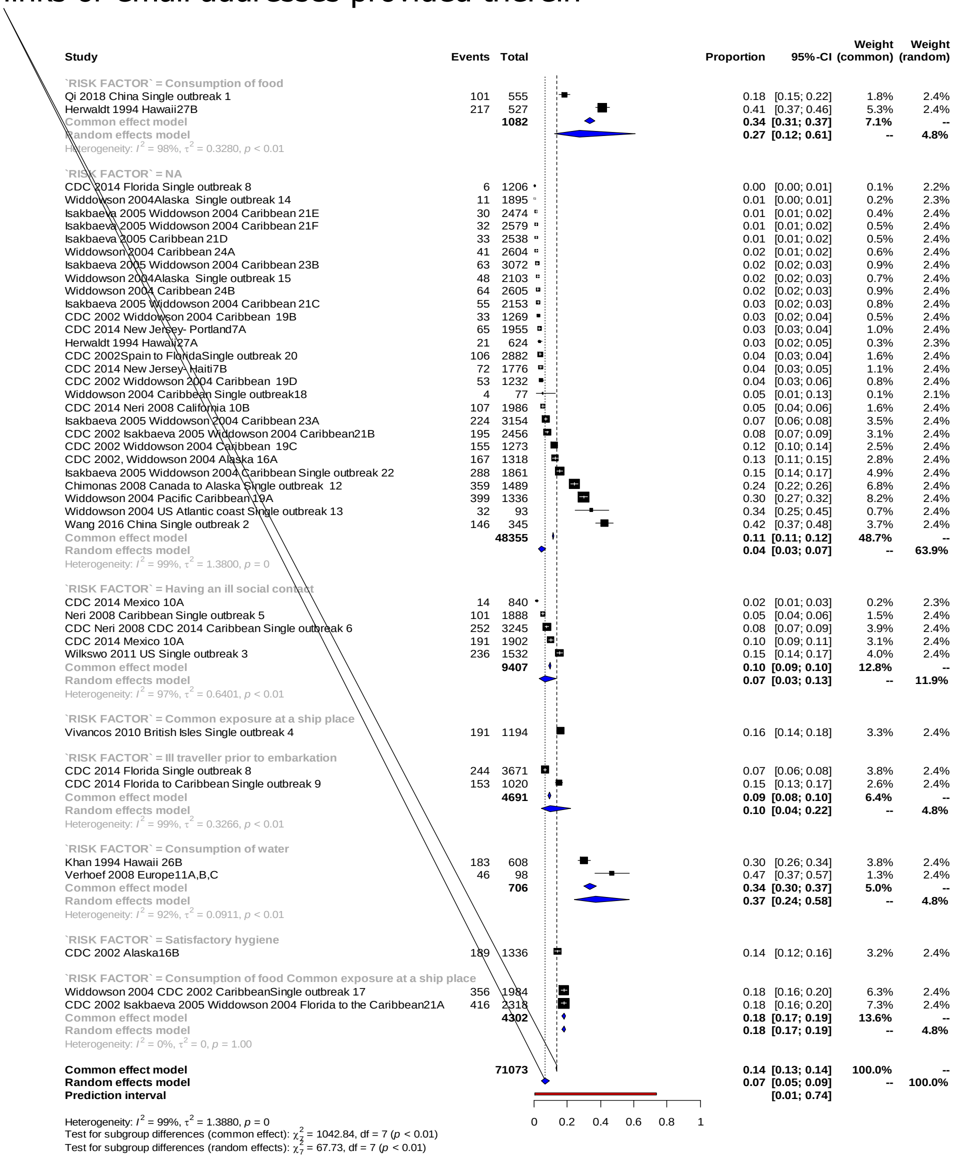

Figure S6: Forest plot: Subgroup analysis for passengers' risk factors, proportions with their corresponding 95% confidence intervals, associated with Norovirus gastroenteritis on cruise ships

This supplementary material is hosted by Eurosurveillance as supporting information alongside the article [A systematic literature review and meta-analysis on preventing and controlling norovirus outbreaks on cruise ships: calling for behaviour change strategies of travellers, 1990-2020], on behalf of the authors, who remain responsible for the accuracy and appropriateness of the content. The same standards for ethics, copyright, attributions and permissions as for the article apply. Supplements are not edited by Eurosurveillance and the journal is not responsible for the maintenance of any links or email addresses provided therein

Study	Events	Total	Proportion	95%-CI	Weight (common)	Weight (random)
`Risk factors` = NA						
CDC 2014 New Jersey- Portland7A	1	978	0.00	[0.00; 0.01]	0.2%	1.9%
Isakbaeva 2005 Widdowson 2004 Caribbean 21F	2	1024	0.00	[0.00; 0.01]	0.4%	2.4%
Widdowson 2004 Caribbean 24A	2	821	0.00	[0.00; 0.01]	0.4%	2.4%
Widdowson 2004Alaska Single outbreak 14	3	874	0.00	[0.00; 0.01]	0.6%	2.7%
Isakbaeva 2005 Caribbean 21D	4	1000	0.00	[0.00; 0.01]	0.8%	2.8%
Isakbaeva 2005 Widdowson 2004 Caribbean 21C	6	1026	0.01	[0.00; 0.01]	1.2%	3.0%
Isakbaeva 2005 Widdowson 2004 Caribbean 21E	6	1020	0.01	[0.00; 0.01]	1.2%	3.0%
Widdowson 2004Alaska Single outbreak 15	7	850	0.01	[0.00; 0.02]	1.4%	3.1%
CDC 2002 Widdowson 2004 Caribbean 19B	7	596	0.01	[0.00; 0.02]	1.4%	3.1%
Widdowson 2004 Caribbean 24B	10	821	0.01	[0.01; 0.02]	2.0%	3.2%
CDC 2014 New Jersey- Haiti7B	13	967	0.01	[0.01; 0.02]	2.6%	3.2%
CDC 2002, Widdowson 2004 Alaska 16A	9	564	0.02	[0.01; 0.03]	1.8%	3.1%
CDC 2002 Isakbaeva 2005 Widdowson 2004 Caribbean21B	23	999	0.02	[0.01; 0.03]	4.7%	3.3%
Isakbaeva 2005 Widdowson 2004 Caribbean 23B	31	1181	0.03	[0.02; 0.04]	6.4%	3.3%
CDC 2002Spain to FloridaSingle outbreak 20	25	944	0.03	[0.02; 0.04]	5.1%	3.3%
CDC 2014 Neri 2008 California 10B	24	844	0.03	[0.02; 0.04]	4.9%	3.3%
CDC 2002 Widdowson 2004 Caribbean 19D	16	562	0.03	[0.02; 0.05]	3.3%	3.2%
CDC 2002 Widdowson 2004 Caribbean 19C	18	586	0.03	[0.02; 0.05]	3.7%	3.3%
Wang 2016 China Single outbreak 2	1	32	0.03	[0.00; 0.16]	0.2%	1.9%
Isakbaeva 2005 Widdowson 2004 Caribbean Single outbreak 22	28	868	0.03	[0.02; 0.05]	5.8%	3.3%
Isakbaeva 2005 Widdowson 2004 Caribbean 23A	39	1184	0.03	[0.02; 0.04]	8.1%	3.3%
Widdowson 2004 Pacific Caribbean 19A	23	600	0.04	[0.02; 0.06]	4.8%	3.3%
Widdowson 2004 US Atlantic coast Single outbreak 13	6	36	0.17	[0.06; 0.33]	1.4%	3.1%
Widdowson 2004 Caribbean Single outbreak18	7	35	0.20	[0.08; 0.37]	1.8%	3.1%
**Common effect model**		**18412**	**0.02**	**[0.02; 0.03]**	**64.4%**	**--**
**Random effects model**			**0.02**	**[0.01; 0.03]**	**--**	**71.8%**
Heterogeneity: $I^2 = 88\%$, $\tau^2 = 1.2046$, $p < 0.01$						
`Risk factors` = Having an ill social contact						
Wilkswo 2011 US Single outbreak 3	7	859	0.01	[0.00; 0.02]	1.4%	3.1%
CDC Neri 2008 CDC 2014 Caribbean Single outbreak 6	20	1183	0.02	[0.01; 0.03]	4.1%	3.3%
**Common effect model**		**2042**	**0.01**	**[0.01; 0.02]**	**5.5%**	**--**
**Random effects model**			**0.01**	**[0.01; 0.03]**	**--**	**6.3%**
Heterogeneity: $I^2 = 64\%$, $\tau^2 = 0.1709$, $p = 0.09$						
`Risk factors` = Common exposure at a ship place						
Vivancos 2010 British Isles Single outbreak 4	5	520	0.01	[0.00; 0.02]	1.0%	2.9%
`Risk factors` = Ill traveller prior to embarkation						
CDC 2014 Florida to Caribbean Single outbreak 9	50	690	0.07	[0.05; 0.09]	10.8%	3.4%
`Risk factors` = Consumption of water						
Verhoef 2008 Europe11A,B,C	2	28	0.07	[0.01; 0.24]	0.4%	2.5%
Khan 1994 Hawaii 26B	19	64	0.30	[0.19; 0.42]	5.4%	3.3%
**Common effect model**		**92**	**0.27**	**[0.19; 0.38]**	**5.8%**	**--**
**Random effects model**			**0.17**	**[0.04; 0.66]**	**--**	**5.8%**
Heterogeneity: $I^2 = 75\%$, $\tau^2 = 0.7641$, $p = 0.04$						
`Risk factors` = Satisfactory hygiene						
CDC 2002 Alaska16B	30	571	0.05	[0.04; 0.07]	6.3%	3.3%
`Risk factors` = Consumption of food Common exposure at a ship place						
Widdowson 2004 CDC 2002 CaribbeanSingle outbreak 17	13	941	0.01	[0.01; 0.02]	2.6%	3.2%
CDC 2002 Isakbaeva 2005 Widdowson 2004 Florida to the Caribbean21A	17	988	0.02	[0.01; 0.03]	3.5%	3.3%
**Common effect model**		**1929**	**0.02**	**[0.01; 0.02]**	**6.1%**	**--**
**Random effects model**			**0.02**	**[0.01; 0.02]**	**--**	**6.5%**
Heterogeneity: $I^2 = 0\%$, $\tau^2 = 0$, $p = 0.55$						
**Common effect model**		**24256**	**0.03**	**[0.03; 0.03]**	**100.0%**	**--**
**Random effects model**			**0.02**	**[0.01; 0.03]**	**--**	**100.0%**
**Prediction interval**				**[0.00; 0.20]**		

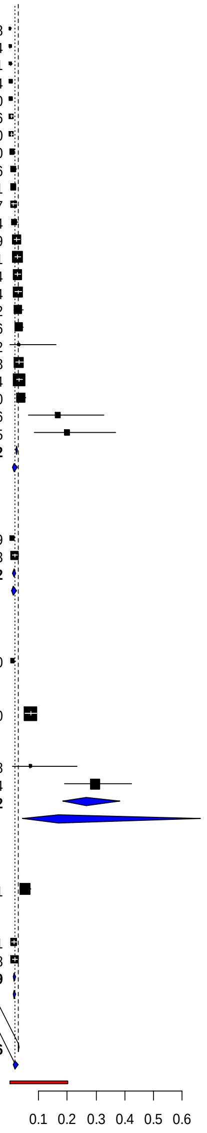

Heterogeneity: $I^2 = 93\%$, $\tau^2 = 1.2781$, $p < 0.01$
Test for subgroup differences (common effect): $\chi^2_6 = 238.86$, df = 6 ($p < 0.01$)
Test for subgroup differences (random effects): $\chi^2_6 = 85.45$, df = 6 ($p < 0.01$)

Figure S7: Forest plot: Subgroup analysis for crew members' risk factors, proportions with their corresponding 95% confidence intervals, associated with Norovirus gastroenteritis on cruise ships
